# Physical characterization and biodegradation of fibers produced by melt-spinning of aliphatic polyesters

**DOI:** 10.1038/s41598-025-04258-z

**Published:** 2025-07-08

**Authors:** Mohammadreza Naeimirad, Bas Krins, Angus McLuskie, Maximilian Vos, Gert-Jan M. Gruter

**Affiliations:** 1 Senbis Polymer Innovation B. V., Eerste Bokslootweg 17, 7821 AT Emmen, The Netherlands; 2 Industrial Sustainable Chemistry, HIMS, Universiteit Van Amsterdam, Science Park 904, 1098 XH Amsterdam, The Netherlands; 3 Avantium N.V., Zekeringstraat 29, 1014 BV Amsterdam, The Netherlands; 4 NHL Stenden University of Applied Sciences, Van Schaikweg 94, 7811 Emmen, The Netherlands

**Keywords:** Microplastics, Aliphatic polyesters, Biodegradable fibers, Sustainability, Melt-spinning, Mechanical performance, Chemical engineering, Sustainability, Environmental sciences, Sustainability

## Abstract

The increasing environmental concerns surrounding synthetic fibers, particularly their contribution to microplastic pollution are driving research toward sustainable alternatives. This study explores the processing, thermal, mechanical, and environmental characteristics of melt-spun biodegradable fibers derived from various biodegradable polymers, including polylactic acid (PLA), polyglycolic acid (PGA), polyhydroxyalkanoates (PHA), polybutylene succinate (PBS), polycaprolactone (PCL), and some copolymers. The melt-spinning process was optimized to address challenges such as thermal degradation, low melt strength, and crystallization limitations. The resulting fibers were analyzed for their mechanical properties, thermal behavior, and biodegradation potential under different environmental conditions, including composting and weathering assessments. The findings indicate that fiber performance is highly dependent on the polymer structure and processing parameters, with PLA and PGA demonstrating superior tensile properties and crystallization behavior, whereas PHA and most copolymers exhibited processing limitations or low-tenacity fibers. The results provide some insights into the development of high-performance biodegradable fibers suitable for textile and technical applications, paving the way for sustainable alternatives to conventional synthetic fibers.

## Introduction

Plastics have become indispensable in modern society due to their versatility, durability, and affordability. However, their widespread use has led to alarming levels of environmental pollution. Between 1950 and 2015, an estimated 6300 million tons of plastic waste were generated, of which approximately 79% remains unprocessed and contributes to environmental pollution^1^. Microplastics, including those originating from textile fibers, have infiltrated aquatic ecosystems, posing risks to marine life and human health^2^. Single-use plastics are a major contributor to pollution, and they account for a significant portion of marine debris. The European Commission estimates that 1.5% to 4% of global plastics produced enter the oceans annually^3,4^. Furthermore, plastic production consumes approximately 4% to 8% of global oil resources, and plastic production figures are projected to triple by 2050, considering most of the oil will go for plastic production, if there is no change in the feedstock^5^. These challenges highlight the urgent need to transition from conventional, fossil-based plastics to bio-based and CO_2_-based, and biodegradable alternatives.

The increasing demand for sustainable and environmentally friendly materials has driven significant research efforts toward the development of biodegradable polymers, particularly for applications in fiber production. The global plastic crisis, fueled by the extensive production and improper disposal of conventional plastics, has created an urgent need for alternatives that reduce environmental impact. Among various approaches, biodegradable fibers produced via melt-spinning are starting to emerge as a promising solution, particularly for replacing polyethylene terephthalate (PET) fibers known as polyester. This introduction outlines the relevance of biodegradable fibers, the critical role of melt-spinning as a fabrication technique, and the key factors influencing the thermal, mechanical, and environmental performance of these fibers^6^.

Biodegradable polymers, especially bio-based aliphatic polyesters offer a dual advantage: they mitigate greenhouse gas emissions by reducing reliance on fossil fuels and provide environmentally benign end-of-life pathways, such as biodegradation. Notable examples include polylactic acid (PLA), polyglycolic acid (PGA), polyhydroxyalkanoates (PHAs e.g. poly(3-hydroxybutyrate) (P3HB) and poly(4-hydroxybutyrate) (P4HB)), polybutylene succinate (PBS), polycaprolactone (PCL), thermoplastic starch (TPS), and copolymers such as polylactic-co-glycolic acid (PLGA), polybutylene adipate-*co*-terephthalate (PBAT), polybutylene succinate-*co*-adipate (PBSA), polybutylene succinate-*co*-terephthalate (PBST), polybutylene sebacate-*co*-terephthalate (PBSeT), polybutylene succinate-*co*-butylene adipate-*co*-ethylene succinate-*co*-ethylene adipate (PBEAS) and PHA copolymers (P3HB4HB, (Poly(3-hydroxybutyrate-co-4-hydroxybutyrate)), PHBV (Poly(3-hydroxybutyrate-co-3-hydroxyvalerate)), and PHBH (Poly(3-hydroxybutyrate-co-3-hydroxyhexanoate)) with different comonomer ratios (more details with the chemical structures are reported elsewhere^6^). These polyesters have garnered interest for their potential to replace conventional plastics with sustainable alternatives in applications ranging from packaging to textiles. Biodegradable polymers are particularly relevant in the textile industry, where fibers represent a significant share of plastic production. Melt-spun biodegradable fibers combine the advantages of scalability and environmental sustainability, making them a compelling alternative to traditional synthetic fibers like PET^6,7^.

Melt-spinning is a widely-used method for producing (biodegradable) man-made fibers. The process involves melting polymer pellets or chips, extruding them through a spinneret, and drawing the resultant filaments to enhance their mechanical properties. Melt-spinning is considered more environmentally friendly than other spinning techniques, such as wet-spinning, which relies on solvents. Key advantages of melt-spinning include scalability, economic viability due to its solvent-free nature and high production speeds, and versatility to accommodate various polymer types, enabling the production of monocomponent, bicomponent, and composite fibers^6,8^. Despite these benefits, challenges such as thermal degradation, low crystallization rates, and limited mechanical performance must be addressed to optimize the process for biodegradable polymers^6,9^.

Different biodegradable polymers exhibit unique properties that influence their processing and end-use performance. For instance, PLA offers high crystallinity and favorable mechanical properties but exhibits very slow environmental biodegradation^10^ or requires specific conditions for composting^11^. PHAs, derived from microbial fermentation, are biodegradable in marine environments but face challenges related to thermal stability and crystallization/solidification rate^12^. PBS and PCL exhibit excellent flexibility and processability but have lower tensile strength and melting points compared to PLA. The properties of melt-spun fibers depend on both the intrinsic characteristics of the polymer and the processing parameters. Key factors include: thermal stability (to withstand the high temperatures involved in melt-spinning without significant degradation), adequate molecular weight (to ensure sufficient melt strength and prevent filament breakage during extrusion), crystallinity, and chain orientation (which influence mechanical properties such as tensile strength, elasticity, and biodegradation rates), additives/modifications, and comonomers/blends (to enhance processability and tailor the properties of the resultant fibers)^6,13^.

Thermal properties, such as glass transition (T_g_) and melting temperature (T_m_), play a critical role in determining the processability and stability of biodegradable fibers. For example, PLA has a high Tm of around 160–180 °C, enabling it to maintain performance for some applications. Conversely, the lower Tm of PCL (~ 60 °C) allows for processing at lower temperatures but limits its high-temperature applications. Mechanical performance, including tensile strength and elongation at break, is equally important. PLA fibers, for instance, can achieve tensile strengths of up to 500 MPa with appropriate drawing techniques. In contrast, PHAs and TPS have critical limitations for fiber melt-spinning processes, such as thermal degradation, low melt strength, and low crystallization rates. PHAs require modifications, such as blending with other polymers or the incorporation of nucleating agents, to achieve processability for spinning and acceptable performance. Additionally, some copolymers like PBAT and PBSA exhibit issues such as low melt strength and high elasticity, which are problematic for the melt-spinning process of multifilament yarns. Our recent review paper reports some recent studies, challenges, and results in this topic^6^. So far, there is no single study or report to give an overall picture of the processing of different biopolymers into fibers and their thermo-mechanical characterization along with disintegration/biodegradation, to understand the effect of the structure of polymers and processing parameters on mechanical properties and biodegradation/composting of melt-spun fibers, in correlation with the environmental conditions which is described schematically^6^. For biodegradable fibers, biodegradation can be in contradiction with other properties in the yarn level such as mechanical properties and durability, and this is especially important for textile applications. Therefore, it is necessary to study the correlation between all of these aspects. Such a study could result in selecting the right polymer or designing a new polymer for developing biodegradable, and also durable fibers for different applications, particularly for textiles.

Melt-spun biodegradable fibers represent a significant step toward addressing the global plastic crisis. However, the commercialization of these fibers faces several challenges, including barriers to polymer processing, higher costs, limited mechanical performance, and regulatory hurdles. However, advancements in polymer chemistry, processing technologies, legislation and policies, and life cycle assessment (LCA) methodologies offer opportunities to overcome these barriers. Key areas for research include promoting circularity, developing cost-effective polymers, optimizing the melt-spinning parameters, enhancing the mechanical and thermal properties of biodegradable fibers, investigating the long-term environmental impact of biodegradable polymers, and scaling up production while maintaining sustainability metrics. Therefore, this report aims to address the correlation between the chemical structure of aforementioned aliphatic (*co*)polyesters, and processing parameters, with thermal and mechanical properties of melt-spun fibers to achieve desired performance and end-of-life scenarios for a proposed sustainable alternative to conventional fibers/yarns with textile or technical applications.

## Experimental

### Materials

Different commercial (the commercial code of these polymers is provided, except for some that were not commercially available or that were synthesized during the project.) biodegradable thermoplastic polymers e.g. PLA, PGA, PBS, PHA, PCL, and copolymers like PLGA, PBAT, PBSA, PBSeT, PBEAS, etc. have been used in this study to perform melt-spinning trials and investigate mechanical performance of melt-spun multifilament yarns with particular emphasis on tenacity and durability. The details about the polymers are provided in Table 1. Furthermore, spin finish Fasavin TC 171 was purchased from Zschimmer & Schwarz GmbH for use in the spinning processes. The ingredients for mesocosm composting tests were obtained according to the standard ISO 20200^17^.

**Table 1:** The materials used in this study.

Polymer	Commercial code	Supplier	MW^a^, IV^b^, or RV^c^	Melt point	Remarks
PLLA	Luminy L175	Total Corbion	MW 175000	177 °C	Semi-crystalline
PLDLA	Ingeo 4032	NatureWorks	MW 180000	168 °C	98% L, 2% D Semi-crystalline
PLDLA	Ingeo 4060	NatureWorks	MW 190000	N/A	92% L, 8% D Amorphous
PBS	FZ91	MCPP	MFI^d^ 5	115 °C	50% bio-base
PCL	Capa 6500	Ingevity	MW 50000	60 °C	
PGA	Kuredux	Kureha	MW 100000	235 °C	
PLGA	N/A	In house	MW 155000	187 °C	89.7% G and 10.3% L content
P3HB	Y3000P	Enmat	–	173 °C	
P3HB4HP	A1000P	CJ Biomaterials	–	N/A	50% 3HB, 50% 4HB Amorphous
PBSA	FD92	Tianjin	MFI 4	85 °C	50% B, 25% S, and 25% A
PBSeT	Ecopond A300	Kingfa	MFI 1.5	115 °C	50% B, 25% Se, and 25% T
PBEAS	BG9000	Anphat-AnTien	MFI 2–5	115 °C	N/A
PBAT	Ecoworld	Jinhui Zhaolong	MFI < 5	115 °C	50% B, 25% A, and 25% T
PET (Ref)	RT5140	Invista	IV 1.02	262 °C	

^a^Molecular weight

^b^Intrinsic viscosity

^c^Relative viscosity

^d^Melt flow index at 190 °C, 2.16 kg

The PLGA was synthesized via a Ring Opening Polymerization (ROP) of lactide and glycolide with a target monomer ratio of 10:90, according to an up-scaled version of the procedure employed by Valderrama et.al.^14^. The synthesis was carried out in a 200L melt-stage polycondensation reactor with 58,515.06g total feed of reagents, which consisted of glycolide (50,000g) and lactide (8500g) monomers, in addition to a tin octanoate (8.67g) catalyst and 1-dodecanol (6.39g) initiator. The reactor was pre-dried for 1 h under vacuum (1 mbar) prior to use. The monomers, catalyst and initiator were added under nitrogen, and stirring (75 RPM) was started once the reagents had fully melted (95 °C). The heating was gradually increased to a reaction temperature of 205–217 °C. The reaction was continued under nitrogen for a further 50 min after no further torque increase was observed. The polymer was discharged via water slide granulation and cut into pellets. The PLGA pellets were dried under vacuum in a tumble drier overnight at 40 °C.

### Melt-spinning

**Fig. 1:**
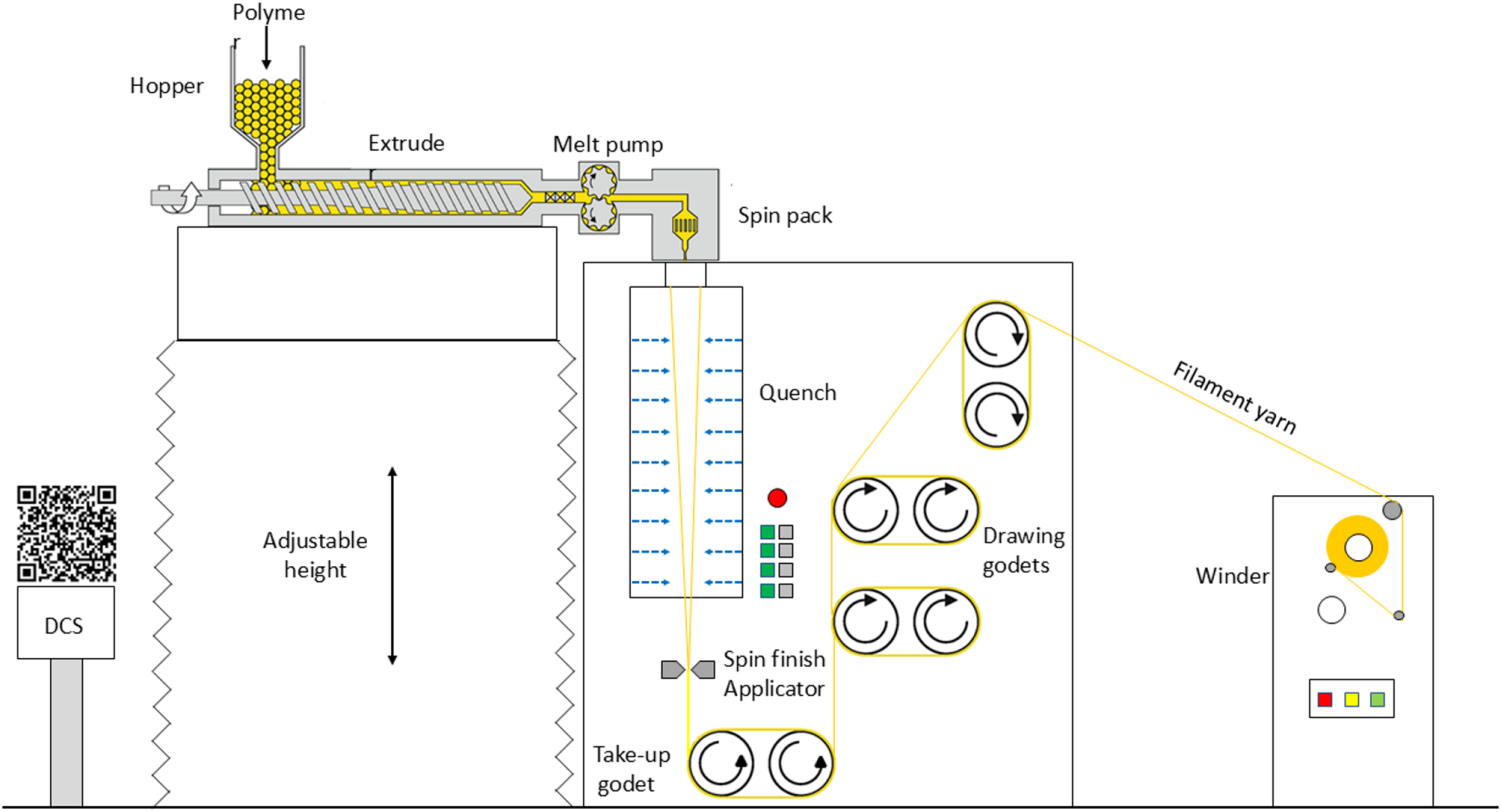
Schematic view of the melt-spinning pilot line at Senbis (Scan the QR code to see the video)^13^.

A versatile bicomponent spinning-drawing-winding (SDW) pilot machine originally built by Fiber Extrusion Technology (FET, Leeds, United Kingdom), with adjustable height for the extruders and 2 floors of quench cabinets (4 m height) has been used for making multifilament yarns out of the polymers (all the polymers were dried in a vacuum dryer for 16 h at the right temperature of 30, 80, 100, or 140 °C, according to the melting point of the polymer). A schematic view of the melt-spinning pilot line is shown in Fig. 1. The smaller extruder with a maximum throughput of 10 kg/h with screw Ø32 mm, L/D (Length to Diameter (known as aspect ratio).) of 30, and a compression ratio of 2.0 was used along with a gear pump with a capacity of 3 cc/rev. The bigger extruder (The maximum throughput of the bigger extruder is 40 kg/h, thanks to the screw with 50 mm in diameter, coupled with a gear pump that can be run with the capacity of 5 or 20 cc/rev.) (up to 40 kg/h) can be used for bicomponent fibers and filaments spinning or up-scaled trials. The used spinneret(s) had 48 orifices with round cross-sections, 400 microns diameter, and L/D of 1.6, while 4 layers of metal net filters with the mesh of 40, 100, 325, 100, and 40 were applied in the spin pack. The spun yarn was passed through the spin finish applicator, before being introduced to the take-up roller and going to the next dues for drawing (orientation) and winding via a Sahm winder (maximum speed of 2500 m/min). All the processing parameters e.g. extruder zone’s temperatures, godets speeds, etc. were adjusted according to the polymer behavior to have a stable process (pressure in the spin pack, filaments in the spin line, take-up speed versus melt strength, and drawability, and finally winding on the spool).

### Characterization

Melt-spun yarns were assessed from different perspectives to get a multidisciplinary understanding of the physical performance of the yarns (mostly linear density, tenacity, and elongation), thermal characteristics (crystallization, etc.), and disintegration (hydrolysis).

### Thermal analysis

Thermal properties (melting and crystallization) of some selective polymers and fiber samples were identified by Differential Scanning Calorimetry (DSC 823e, Mettler Toledo AG, Switzerland) at the rate of 10 °C/min from 25 to 250 °C through continuous heating and cooling cycles.

### Optical microscopy

Cross-sections of the fibers were visually investigated using an optical microscope Olympus BH-2, (Olympus Corporation, Japan) with 20–100 × magnification, utilizing normal light. Furthermore, the measured diameters can be correlated to the liner density or DPF (Denier Per Filament.) of the fibers (knowing the round shape spinneret, and the density of the polymer).

### Physical characterization

The linear density of melt-spun yarns was determined by utilizing a Zweigle L232. 50 m of yarn was collected with a tension of 5 g/tex (n = 3). The mass of the yarn was measured on an analytical balance AE260 (Mettler Toledo AG, Switzerland). Also, tensile properties were assessed according to standard ASTM D2256^15^ using a tensile tester model 5564 (Instron, USA)^16^. The tensile tester was equipped with a 1 kN load cell and pneumatic yarn grips. A crosshead speed of 250 mm/min and a gauge length of 250 mm were used. Each specimen was measured 5 times (n = 5) with a pretension of 5 mN/tex and a yarn twist of 60 TPM (Turns Per Meter.)

### Weathering

Some selective yarns (PLA and PGA) were hung/aged in the lab environment (temperature of 25 °C and relative humidity of 65%) to see the effect of weathering (without UV) on the physical properties of the yarns. Mechanical properties of the yarns were assessed periodically during the test while the molecular weight of the samples was measured at the end of the test using Gel Permeation Chromatography (GPC). GPC measurements were carried out on a Hitachi Chromaster 5450 with an Agilent HPLC system equipped with two PFG 7 µm (μm) Linear M (300 × 7.5 mm) columns.

 HFIP was used as mobile phase with a 1mL/min flow and T = 35 °C. Calculation of the molecular weights were carried out with Astra 6 Software. Furthermore, mesocosm studies were performed on all melt-spun samples to investigate the disintegration of yarns which is a preliminary step for ultimate biodegradation.

### Mesocosm assessment

Melt-spun yarns were converted to fabric samples via a circular socks knitting machine, 150 mm diameter and gauge 10 per inch. The fabric samples were cut, weighted, and fixed between DIA slide frames (24 mm × 36 mm, Else, Germany) to perform disintegration assessments in different environments (including composting according to ISO 20200^17^). Mesocosm studies have been performed in home composting and industrial composting conditions with temperatures of 28 and 58 °C, respectively. The temperature of the incubator, moisture content of the containers, and the samples were monitored while the samples were assessed visually at periodic times.

## Results and discussion

The initial polymer analysis and the spinning trials resulted in final yarns on the spool for further characterizations, or technical understandings which are elaborated in the following.

### Processability

Over 30 trials were performed to make the appropriate yarns from the aforementioned polymers. Thermal degradation of biodegradable polymers was limiting the processing window (particularly the maximum extrusion temperature for PHAs and PLAs), while polymer melt-strength was challenging for increasing the take-up speed (filaments of co-polymers e.g. PBAT and PBSA had low melt-strength and high elasticity in the spin line). Furthermore, spinning of some polymers (especially PHAs) faced slow solidification and resulted in sticky filaments (proposing different approaches for making multifilament yarns from PHAs). Although most of the filament yarns were successfully introduced to the first godet, more optimizations were needed in the hot godets (speeds for drawing, and temperatures for crystallization). However, the melt spinning of some polymers e.g. PET, PLA, and PGA was quite smooth and with pretty high speed. Table 2 indicates processing parameters for making the best/final sample from each trial e.g. the maximum temperature in extruder zones (processing temperature), the maximum temperature of the godet pairs (set temperature), and the maximum speed (winding speed). The speed of the gear pump can be calculated from the throughput based on the capacity of the pump’s gear (3 cc/rev), while the take-up roller speed can be calculated based on the winding speed and solid drawing ratio.

**Table 2:** The main processing parameters for making the best yarn on the spool from each polymer.

Biopolymer	Spool number	Process temp (°C)	Throughput (cc/min)	Draw ratio	Set temp (°C)	Winding (m/min)	Remarks
PGA FDY	M93-112-FS01	240	30	4.75	120	950	
PGA POY	M93-93 AS08	240	30	2.5	80	1000	
PLGA	M93-142-AS06	210	17.5	5.4	68	700	
PBS (BG)	M93-001-RS	240	15	4	70	1200	
PBS (FZ91)	M93-93-BS07	275	30	2.6	60	650	Drawing problem
PBEAS	M93-171-AS13	240	33	3	60	560	
PBSeT	M93-154-AS04	250	30	2.7	75	525	
PCL	M93-045-AS04	110	45	5	40	980	Thermally stable
PLA	M93-109-FS02	210	24	3.5	120	690	
PLLA	M93-109-CS04	210	24	4.25	115	840	
PLDLA	M93-109-DS01	200	24	3	100	590	
PHA (blend)	M93-108-IS02	185	21	1.8	70	395	
PET	M93-088-AS15	295	18.6	3	100	1140	Reference

The polymers that are not listed here were not able to be spun (at least with high speed SDW).

The results show that the extrusion of biodegradable polymers (particularly for fiber spinning) requires more consideration to avoid exceeded thermal degradation (due to the processing temperature of some of the polyesters being close to their thermal degradation, accelerated with residence time). Furthermore, the homo-polymers e.g. PLA and PGA are better suited for spinning (faster crystallization, PHAs are the exception due to slow crystallization rate), while copolymers e.g. PBAT and PBSA are showing slow crystallization rates, therefore lower melt-strength, and challenging process. Although spinning of PCL and PBS worked out, the rate of crystallization was hampering the solid drawing process, particularly for PBS grades^18^.

The mechanical properties and thermal shrinkage of melt-spun fibers from co-polymers (particularly less crystalline e.g. PLGA or PLDLA) can be questionable. At the same time, the low melting point of PBS and PCL yarns remains a barrier for some applications e.g. textiles.

### Thermal properties

**Fig. 2:**
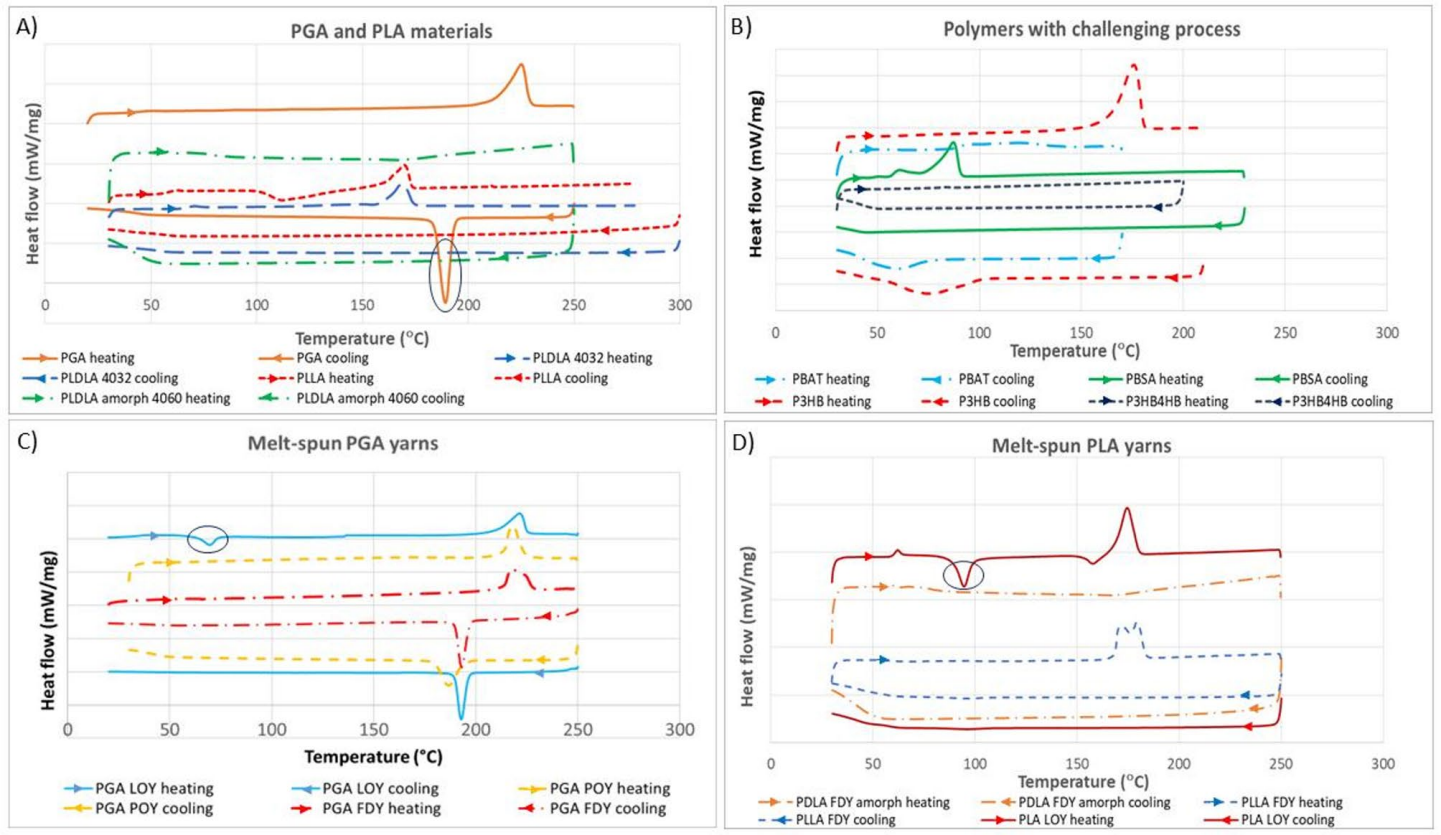
Resulted DSC graphs showing thermal properties of biopolymers and some melt-sun fiber in the heating and cooling cycle: (**A**) PGA and PLA polymers (PLLA, PLDLA); the circle highlights the PGA crystallization), (**B**) some challenging (co)polymers (PBAT, PBSA, and P3HB4HB, along with P3HB), (**C**) PGA yarns (LOY, POY, and FDY); the circle highlights the PGA LOY cold crystallization), and (**D**) PLA fibers (LOY, and FDY from semi-crystalline PLLA, and amorphous PLDLA); the circle highlights the PLA LOY cold crystallization).

Figure 2 represents the resulting graphs of DSC analysis, including heating and cooling curves, for some polymers and fibers.

The DSC analysis on polymers show that PGA has a much faster crystallization rate than PLA (there is no crystallization peak for the cooling curve of PLA). This is confirmed in as-received polymers (panel A). This is attributed to the residence time of the polymer at crystallization temperature and the crystallization rate which is not fast enough in the case of PLA (with 10 °C/min cooling). The DSC results for PGA agree with the crystal perfection of this polymer at high temperatures, as reported elsewhere^19^. Although DSC does not give a good picture of the real behavior during the spinning, it is clear that the residence time of the polymer in the spin line will be less than 1 s, and the high-temperature difference between polymer melt and the quench cabin will be even larger than the DSC conditions. This shows that melt-spun yarns from semi-crystalline polymers e.g. PLA need to be set on the drawing godet by increasing the temperature as close as possible to the crystallization temperature of the polymer. The PLDLA copolymer does not show a melting or crystallization peak as it is fully amorphous by design. Also, it can be concluded from the DSC graphs that copolymers show a broad melting peak and a broad (or in some cases no) crystallization peak (panel B). This is attributed to the molecular disarrangement and the more amorphous nature of the polymers. However, PGA fibers show melting peaks indicating their crystallinity, and re-crystallization during cooling while the Low Oriented Yarn (LOY) sample shows cold crystallization versus Partly Oriented Yarn (POY) and Fully Drawn Yarn (FDY) (panel C). The cold crystallization peak appeared below 100 °C in the heating curve of LOY yarns of both PGA and PLA. This is a piece of evidence that although PGA fibers show crystallization, there is a need for drawing and setting for the best performance. FDY PLA fibers from the semi-crystalline type show a melting peak while there is no peak for amorphous PLA (panel D).

Furthermore, the degree of crystallinity (X_c_) was calculated using DSC data according to the following equation:

$${X}_{c}(\%)=\frac{\Delta {H}_{m}-\Delta {H}_{cc}}{\Delta {H}_{m}^{o}}\times 100$$

ΔH_m_ and ΔH_cc_ are the melting and cold crystallization enthalpies obtained from the DSC curves, and ΔH^o^_m_ is the melting enthalpy of 100% crystalline polymer. The results of the calculations are presented in Table 3.

**Table 3:** Enthalpies obtained from the DSC graphs and the resulting crystallinity (some extra samples additionally).

Sample	ΔH_m_ (J/g)	ΔH_cc_ (J/g)	ΔH^o^_m_ (J/g)	X_c_
PLDLA amorph 4060 (fiber)	0	0	93	0%
PDLA amorph FDY (fiber)	0	0	93	0%
PLDLA 4032 (pellet)	28.53	0	93	30%
PLLA (pellet)	21	0	93	22%
PLLA LOY (fiber)	41.5	2.15	93	42%
PLLA FDY (fiber)	51	0	93	54%
PGA (pellet)	62.3	0	140	44%
PGA LOY (fiber)	78.77	24.96	140	38%
PGA POY (fiber)	79	0	140	55%
PGA FDY (fiber)	85	0	140	60%
PBAT (pellet)	16	0	114	14%
PBSA (pellet)	39.47	0	114	34%
P3HB (pellet)	88.28	0	146	60%
P3HB4HB amorph (pellet)	0	0	146	0%
PBS (pellet)	58.93	0	110	53%
PBS POY (fiber)	68.46	0	110	61%
PCL (pellet)	56.8	0	136	42%
PCL FDY (fiber)	68.61	0	136	50%

According to the results in Table 3, fibers presented higher crystallinity than the counterpart pellets (even FDY more than POY) due to the orientation-induced crystallization, and heat set in the drawing stage. Also, PGA and PBS show higher crystallinity than other biopolymers, which is attributed to their more linear structure, while copolymers have less crystalline structure, and some of them are even fully amorphous.

The correlation between the thermal analysis results and the processing outcomes is that the more regular and crystalline polymer, with a visible crystallization rate (a sharp peak in the cooling curve), the better the spinning process is expected. As mentioned before, PHAs (particularly P3HB) show crystallization peaks in the DSC analysis, while they have limitations in the process due to slow solidification and stickiness of filaments.

### Physical properties

**Fig. 3:**
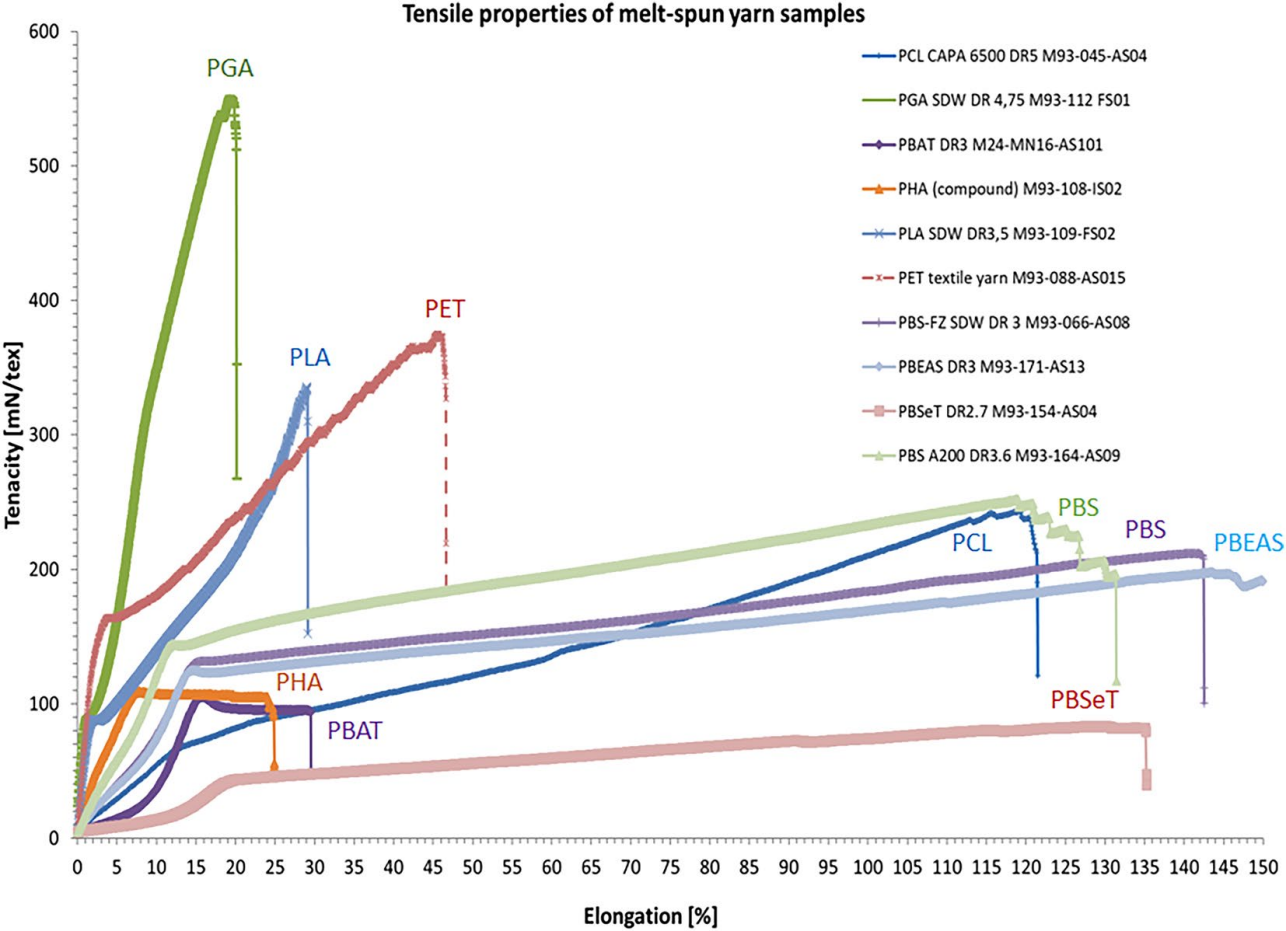
Tensile properties of some selected melt-spun multifilament yarns.

Variation in the melt-spinning trials for some of the polyesters e.g. PLA, PBS, PCL, etc. resulted in the production of different spools. The tensile testing graphs of selected melt-spun yarns are compared in Fig. 3.^20^

As displayed in the figure, different polyesters show different tensile properties (tenacity and elongation measured on the filament), which is correlated with the structure of the polymer (e.g. high regularity, resulting in higher crystallinity), and processing parameters (e.g. drawing ratio or orientation, setting temperatures, and the final winding speed). PLA yarn has quite higher tensile properties compared to the other yarns, particularly as compared to copolymers, while PGA exhibits higher mechanical properties, particularely in tenacity and tensile modulus. The detailed tensile properties of multifilament samples including liner density (LD), breaking force (BF), breaking tenacity (BT), elongation at break (EAB), and tensile modulus (or initial modulus) (TM) are presented in Table 4 with average (AV) and standard deviations (SD).

**Table 4:** Mechanical properties of melt-spun yarns according to the tensile testing results.

Properties	LD		BT		BF		EAB		TM	
Scale	[dtex]		[mN/tex]		[N]		[%]		[mN/tex]	
Sample	AV	SD	AV	SD	AV	SD	AV	SD	AV	SD
PET M93-88-AS015	204.3	1.5	351.2	27.1	7.1	0.5	35.8	8.1	8814.2	273.6
PLA LOY	2494.9	4.5	47.6	5.2	11.8	1.3	240.0	0.1	2408.4	226.2
PLA M93-109-FS02	395.1	0.6	350.3	25.9	13.8	1.0	29.2	0.6	6758.0	161.9
PLDA M93-109-DS01	507.2	9.2	136.7	9.7	6.9	0.5	27.1	0.7	3826.9	224.8
PLAA M93-109-CS04	322.0	3.0	275.9	24.3	8.8	0.8	25.7	1.4	7367.0	336.8
PBS(FZ) M93-93-BS07	590.0	4.2	154.7	4.9	9.1	0.3	252.7	13.3	873.4	71.6
PBS(BG) M93-001-RS	190.8	2.8	197.7	5.9	3.7	0.1	77.8	5.4	1493.2	70.9
PBS M93-164-AS09	486.0	71.1	259.7	11.9	12.6	0.6	122.1	6.7	1493.2	70.9
PGA LOY	2874.6	123	40.3	5.8	11.5	1.6	243.9	7.0	1559.4	159.6
PGA POY M93-93-AS08	297.0	6.0	226.0	17.0	6.7	0.5	45.0	7.0	5500	300.0
PGA FDY M93-112-FS01	385.5	7.4	531.7	30.0	20.5	1.1	19.2	1.1	10,398.5	186.3
PCL(6500) M93-045-AS04	493.0	17.0	250.0	12.0	12–0	1.0	127.0	12.0	635.0	40.0
PLGA M93-142-AS06	360.8	9.5	191.6	27.2	6.9	0.9	38.7	2.8	4152.0	382.8
PBSeT M93-154-AS04	625.0	27.6	86.4	13.6	5.4	0.9	141.2	24.4	130.6	10.9
PBEAS M93-171-AS13	696.3	6.7	192.6	6.3	13.4	0.4	142.8	9.7	847.0	13.7
PHA M93-108-IS02	327.7	11.9	108.3	0.9	3.5	0.1	14.8	16.2	2367.1	302.2

The test results on semi-crystalline PLA agree with other studies, showing 20–60% crystallinity and 200–600 MPa tenacity^6,21^. However, the strength of amorphous PLDLA fibers is much less (around 135 mN/tex) with zero crystallinity, indicating a low mechanical performance typically associated with high thermal shrinkage. The results achieved so far between different grades of PLAs are in the same line with previous reports^22,23^. The spinning results of PHAs show that this is so far only possible by a monofilament approach, or quenching in a water bath, otherwise by blending with other polymers, while mechanical properties stay low according to multiple reported cases^12,24,25^. The trials of PGA and PLGA filament yarns show that the winding speed can be higher, while the application can expand to more than only biomedical cases reported in the literature^6,19,26,27^. Processing parameters and the thermo-mechanical results for PCL yarns are in the same line with other reports^6,28,29^. There is a new window for the technical application of these yarns in low-temperature environments. Although high-speed melt-spinning trials of some co-polymers e.g. PBAT, and PBSA resulted in some challenges like slow crystallization, low melt strength, and elastic behavior, there are other studies showing melt-spinning of these copolymers (regardless of the final speed)^30,31^. Tenacity of PBS yarns is higher than some reports^32^ (this is attributed to the high crystallinity in PBS fibers, even a shish-kebab structure reported elsewhere^18^), while the low tenacity and flexibility of co-polyester fibers are comparable with the other reports^30,32–34^. This can be extended to PHAs (e.g. P3HB4HB or PHBH versus PHB) as well, reported elsewhere^35,36^.

### Morphology

**Fig. 4:**
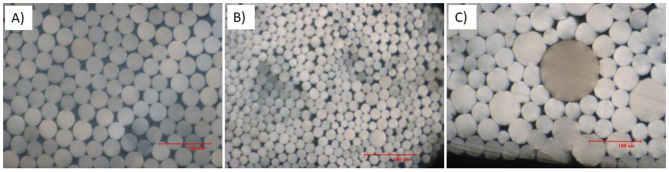
Cross-section of some fibers with optical microscopy: (**A**) PLA yarns (590 dtex), (**B**) PBS yarns (167 dtex), (**C**) PCL yarns (760 dtex).

The optical microscopic images shown in Fig. 4 illustrate round cross-sections for all the melt-spun fibers. However, the round-perfection and diameters are different due to the difference in different factors e.g. viscosity of the polymer, crystallization rate, processing temperature, quench rate, and the final winding speed.

According to Fig. 4 (A) the diameter of PLA filaments with round cross-section is around 30 µm which is in agreement with the count of yarn (590 dtex, and DPF 12). The diameter of PBS filaments (B) is smaller (around 15 µm) which is in agreement with the lower count of the yarn (167 dtex, 3 DPF). The variation of the diameters of the PCL filaments (C) is due to the fast and irregular crystallization, along with low drawing ratio.

### Disintegration

**Fig. 5:**
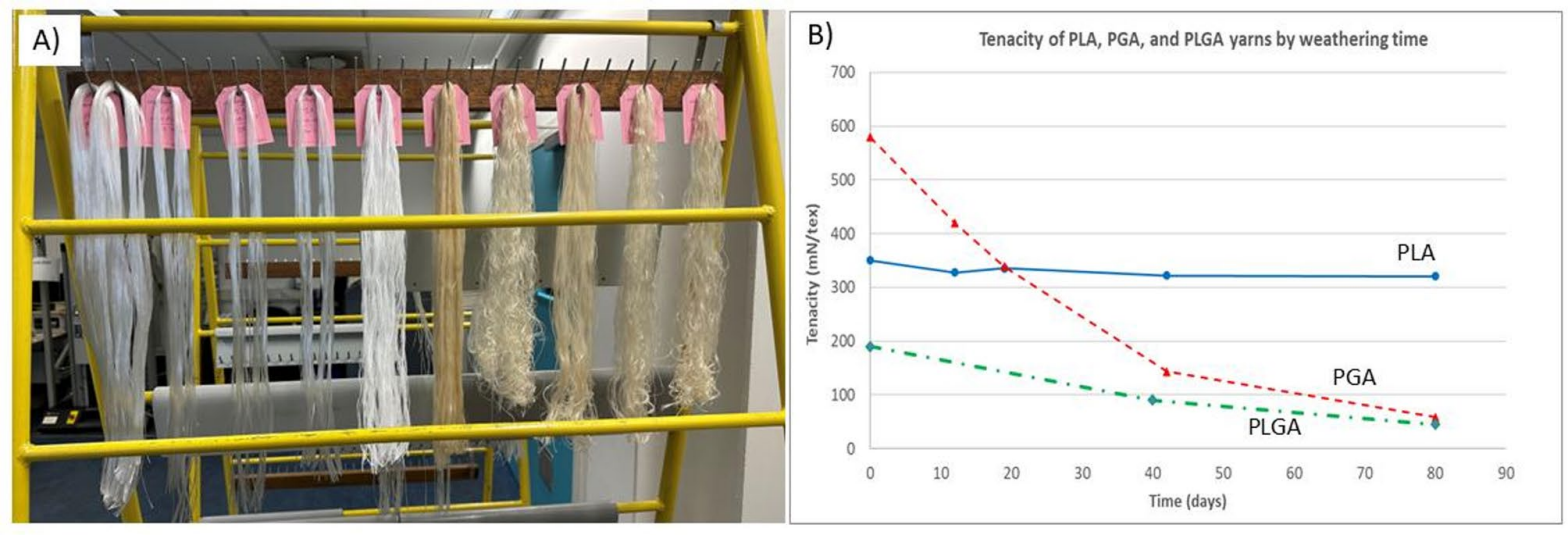
(**A**) Weathering of PLA, PGA, and PLGA yarns in the standard lab environment, and (**B**) Tensile tenacity of the PLA, PGA, and PLGA yarns during the test.

Disintegration is the preliminary observable effect of biodegradation. Several factors impact the disintegration rate, e.g., moisture (abiotic hydrolysis), enzymes (enzymatic hydrolysis), ultraviolet (UV), and initiating radical reactions. These parameters can affect different polymers at different levels. The weathering assessment results (Fig. 5) show that PGA yarns are very sensitive to ambient moisture and lose more than 90% of mechanical properties within 80 days, while PLA yarns perform almost the same after this weathering period. PLGA yarns fall in between with 75% tenacity loss.

The GPC results (Table 5) confirm that the mechanical property drop in PGA yarns during weathering is due to abiotic hydrolysis and chain scission (as there are no enzymes or bacteria in the atmosphere while the MW drops). For any specific polyester, hydrolysis can be accomplished in amorphous areas and in crystalline regions with different rates^37^ which can be considered in the disintegration rate of different yarns e.g. LOY, POY, and FDY. In the case of PGA and PLGA, the reduction in M_W_ values are 97% (80 days) and 90% (110 days) respectively, while for PLA the M_w_ drop is far less, at 8% (80 days). Since this analysis was not done on as-spun yarns for PLA and PGA, the internal experience on other melt-spun polyester yarns is around 8–10% IV or MW drop compared to the polymer (showing that this drop could have resulted also on as-spun PLA yarn). The trend is in agreement with Fig. 5.

**Table 5:** GPC results for the weathered PLA, PGA, and PLGA yarn samples, and as-received polymers.

Sample	Mn (Da)	Mw (Da)	PDI (Mw/Mn)	Mn drop (%)	Mw drop (%)
PLA pellet	88,654	187,150	2.11	15.12	8.37
PLA yarn (after 80 days)	75,245	171,477	2.28		
PGA pellet	76,192	151,376	1.99	97.37	97.44
PGA yarn (after 80 days)	2000	3868	1.93		
PLGA Pellet	46,500	155,000	3.33	–	–
PLGA yarn (new)	34,960	94,120	2.69	86.44	90.03
PLGA yarn (after 110 days)	4742	9382	1.98		

**Fig. 6:**
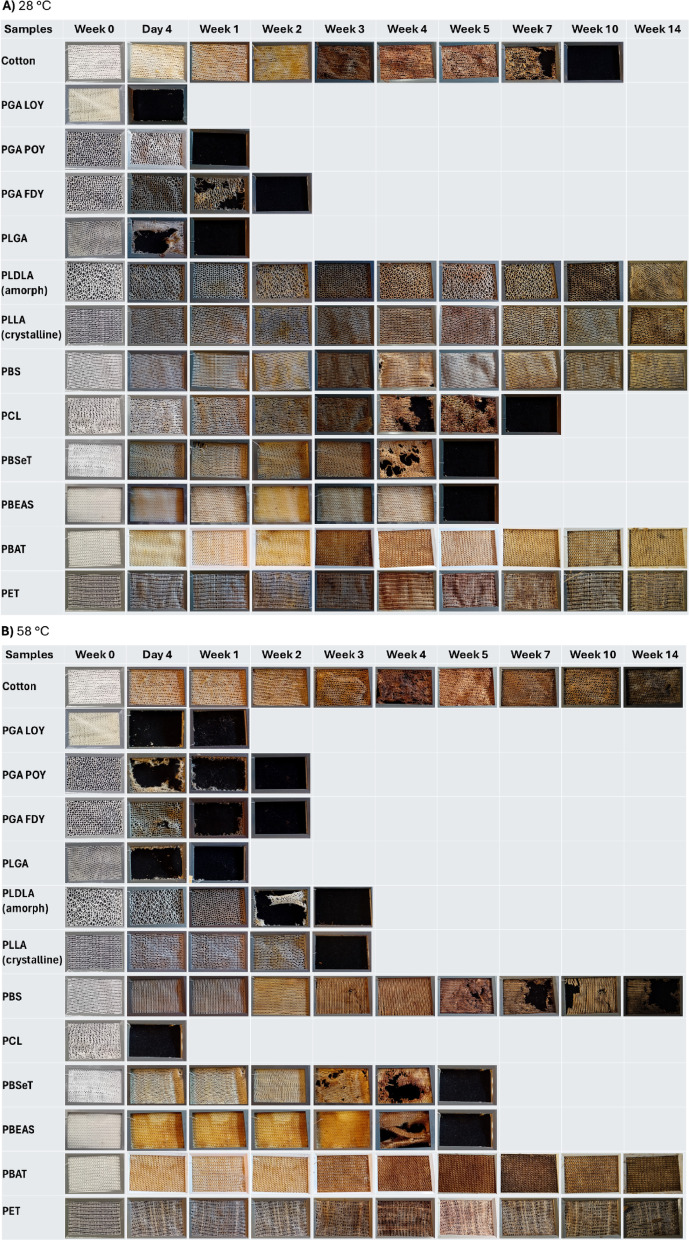
Disintegration rates of knitted fabric samples from various melt-spun yarns during mesocosm assessments: (**A**) home composting (28 °C) and (**B**) industrial composting (58 °C).

The weathering results were a good reason to investigate the disintegration of the filaments (in fabric form) in compost under different conditions. The visual inspection of the fabric samples after some periodic assessments in home composting and industrial composting conditions are shown in Fig. 6.

According to Fig. 6, PGA samples show the fastest composting rate (disintegration within 1 week, especially for LOY and POY samples) at both temperatures. The order of disintegration rate between LOY, POY, and FDY PGA samples and the minor difference with PLGA is due to the difference in crystallinity and also capping of hydroxyl groups (in the case of PLGA) which is in agreement with the results of previous studies^19,38,39^. This shows that PGA is very sensitive to abiotic hydrolysis which is attributed to the accessibility and hydrophilicity of the ester bonds (starting from amorphous regions), although the regular structure of PGA polymer chains is ideal for the formation of crystalline regions (they undergo hydrolysis at a lower rate)^19^. Furthermore, hydrolysis-assisted carboxylic group release accelerates the disintegration rate^26^. However, the biodegradation rate of PGA and PLGA might differ in the ultimate microbial utilization step, and this can be a proposed study for lab assessments, following the report by Wang et al.^10^. Moreover, PCL disintegrated within a week only in industrial composting, which is due to the low melting point (very close to 58 °C). However, the counterpart sample disintegrated totally in home compost after 7 weeks. PLA samples only disintegrated under industrial composting conditions (after 3 weeks, while home composting samples did not show any disintegration up to 14 weeks). Amorphous PLA disintegrates faster than semi-crystalline PLA. This is due to the different hydrolysis rates in amorphous and crystalline areas^40^. Furthermore, the less crystalline the polymer structure for each given polymer, the faster the disintegration and probably the final biodegradation.

Further investigations on the other fibrous samples show that PBS is disintegrated in industrial composting (not in home compost, at least up to 14 weeks). However, some copolymers e.g. PBEAS and PBSeT are disintegrated even under home composting conditions (within 4 weeks) which is in agreement with other studies^33^. PBAT does not show any disintegration, not even under industrial composting conditions. This is attributed to the effect of chemical structure (especially in (co)monomer selection, and (co)polymer design: The more disarrangement e.g. PBEAS, the less crystalline structure, resulting in faster disintegration. At the same time it can also be concluded that the more stable the bonds/chemicals e.g. using terephthalic acid in PBAT, the less disintegration rate).

Although the stability of PET is well known (as PET is conventionally non-degradable), cotton is a (bio)degradable fiber in some conditions. The results show a lower disintegration rate for cotton at the higher temperature of the industrial composting experiments (no major disintegration up to 14 weeks), while the cotton samples under home composting conditions are completely disintegrated within 10 weeks. This is attributed to the hydrolysis pathway of polymers (abiotic or enzymatic) to be ready for biodegradation. Although the high temperature accelerates abiotic hydrolysis (in case of PGA, PLA, PBS, and PCL), it affects microorganisms activity responsible for the hydrolysis (limiting growth of hydrolase enzymes), lowering enzymatic hydrolysis for certain polymers such as cellulose and PHAs.

These findings suggest an idea for optimized temperature e.g. 37 °C (simulated from the human body, appropriate for biological activities) for accelerated hydrolysis and biodegradation assessments. Furthermore, the next paper will report on the ultimate (aerobic) biodegradation (in soil and wastewater) of different (co)polymers (including several types of PHAs) with particular emphasis on hydrolysis pathways along with the discussion on the effect of the chemical backbone and morphological structure of polymers on both hydrolysis and microbial utilization.

**Fig. 7:**
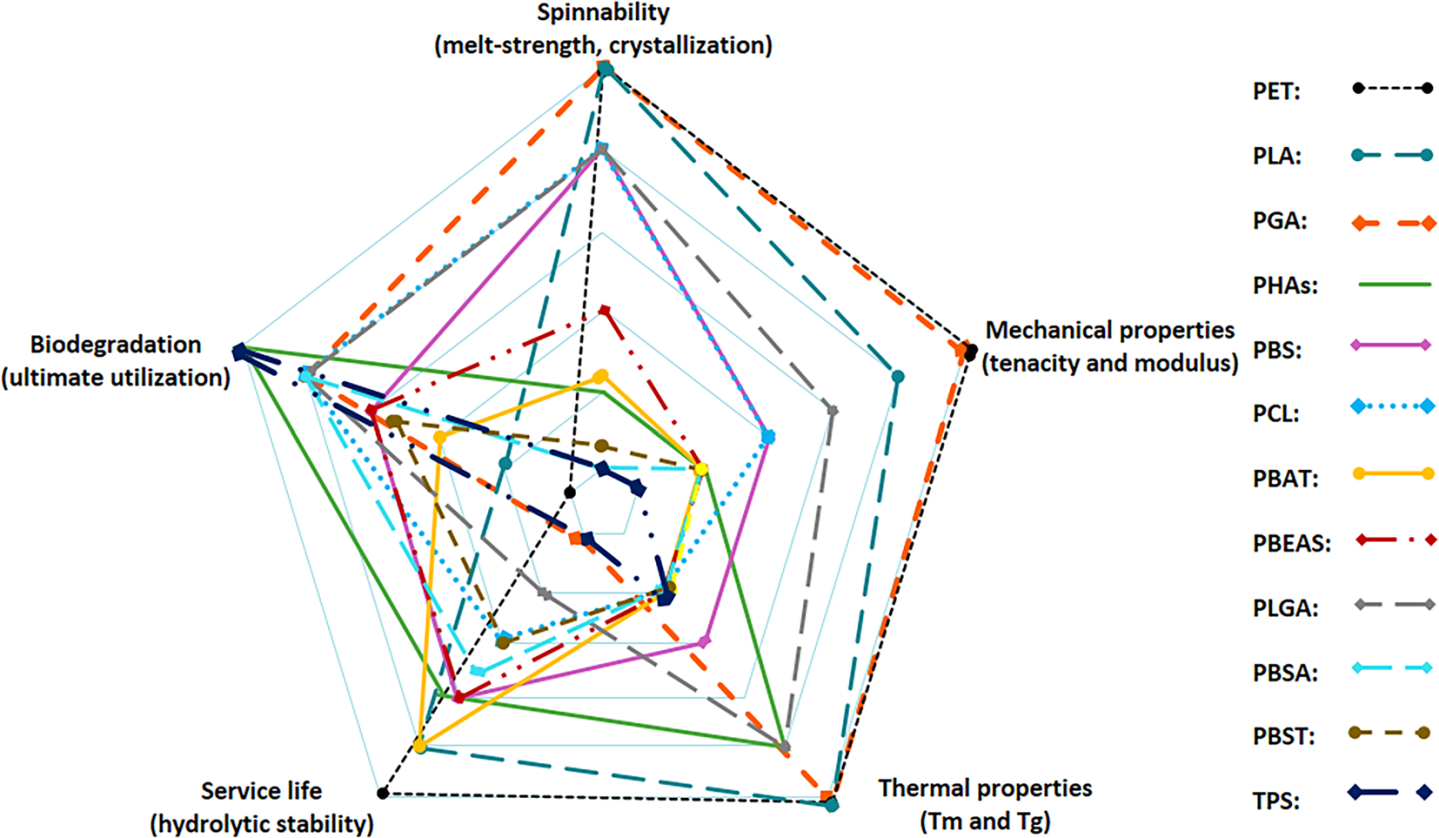
Overall view of different performance aspects of biodegradable polyester fibers (processability, mechanical properties, thermal properties, service life, and biodegradation/disintegration rate) which all result from the chemical backbone and morphology of the polymer, and the processing parameters.

Figure 7 underscores different aspects of biodegradable polymers in fiber melt-spinning, illustrating that no single polymer excels across all the boxes, necessitating careful selection based on the intended application (if we consider the hydrolysis or biodegradation rate on the opposite side of service life or durability).

## Conclusion

This study demonstrates that melt-spun biodegradable polyester fibers present viable alternatives for conventional synthetic fibers, and their performance is dictated by processability, mechanical strength, thermal stability, hydrolysis rate, and biodegradation behavior. These differences can be attributed to the chemical structure of the polymers, which influences their crystallinity, rate of crystallization, and melt strength, among other factors. For instance, PLA and PGA exhibit high spinnability and tensile strength, but PGA disintegrates rapidly in humid conditions, limiting its durability or service life. PHAs show excellent biodegradability, and even surpass cellulose, but PHAs suffer from slow crystallization and low melt strength, making high-speed spinning challenging. Copolymers like PBAT and PBSA offer greater flexibility but exhibit lower melt strength for spinning, and ultimately lower mechanical properties. These findings highlight the critical need to balance mechanical performance, durability, and environmental impact when developing biodegradable fibers for textile/industrial applications, and even when developing new polymers or compounds.

Furthermore, a clear outcome is that the chemical backbone of aliphatic polyesters e.g. PGA, PLA, and PBS is a determining factor in polymer morphology and characteristics like Tm, Tg, and crystallization, while it can also affect the mechanical properties and hydrolysis/biodegradation rate. The regularity of the structure determines the crystallinity (e.g. PLLA versus PLDLA). This is even extendable to copolymers e.g. PLGA, PBSA, etc., while the structure/property is even manipulatable with the ratio of comonomers and copolymer type.

### Outlook

Recent reports and changing legislation show that the area of biodegradable polymers is becoming increasingly interesting, not only from the academic point of view but also from the industrial perspective. Still, we are only at the beginning of the transition from fossil to renewable materials and more work is needed to pave the way for developing biodegradable alternatives for fibrous products. Here are some prospect suggestions:

- Studying biodegradation (final utilization by microorganisms) of polyesters and their fibers with emphasis on the abiotic hydrolysis pathways (marine degradability). It is worth mentioning that a systematic study is underway at Senbis Polymer Innovations and the University of Amsterdam, and the results will be published by the end of 2025.
- Field tests to understand the disintegration/biodegradation of the products under real application conditions which should be considered for any development of sustainable products.
- Designing new (*co*)polymers for fibers with optimized physical performance and environmental aspects for target applications^41,42^.
- Development of technical yarns/filaments, also from biodegradable polymers. Several soil-degradable and/or marine degradable filament products have recently been introduced in the market and more products for a better environment are expected^43^.

## Data Availability

The datasets generated and/or analysed during the current study are not publicly available due to internal rules, but are available from the corresponding author on reasonable request.
